# Targeting the undruggable: exploiting neomorphic features of fusion oncoproteins in childhood sarcomas for innovative therapies

**DOI:** 10.1007/s10555-019-09839-9

**Published:** 2020-01-22

**Authors:** Maximilian M. L. Knott, Tilman L. B. Hölting, Shunya Ohmura, Thomas Kirchner, Florencia Cidre-Aranaz, Thomas G. P. Grünewald

**Affiliations:** 1 Max-Eder Research Group for Pediatric Sarcoma Biology, Institute of Pathology, Faculty of Medicine, LMU Munich, Thalkirchner Str. 36, 80337 Munich, Germany; 2 Faculty of Medicine, Institute of Pathology, LMU Munich, Munich, Germany; 3 German Cancer Consortium (DKTK), partner site Munich, Munich, Germany; 4 German Cancer Research Center (DKFZ), Heidelberg, Germany

**Keywords:** Ewing sarcoma, Synovial sarcoma, Rhabdomyosarcoma, Fusion oncogene, Targeted therapy

## Abstract

While sarcomas account for approximately 1% of malignant tumors of adults, they are particularly more common in children and adolescents affected by cancer. In contrast to malignancies that occur in later stages of life, childhood tumors, including sarcoma, are characterized by a striking paucity of somatic mutations. However, entity-defining fusion oncogenes acting as the main oncogenic driver mutations are frequently found in pediatric bone and soft-tissue sarcomas such as Ewing sarcoma (*EWSR1-FLI1*), alveolar rhabdomyosarcoma (*PAX3/7-FOXO1*), and synovial sarcoma (*SS18-SSX1/2/4*). Since strong oncogene-dependency has been demonstrated in these entities, direct pharmacological targeting of these fusion oncogenes has been excessively attempted, thus far, with limited success. Despite apparent challenges, our increasing understanding of the neomorphic features of these fusion oncogenes in conjunction with rapid technological advances will likely enable the development of new strategies to therapeutically exploit these neomorphic features and to ultimately turn the “undruggable” into first-line target structures. In this review, we provide a broad overview of the current literature on targeting neomorphic features of fusion oncogenes found in Ewing sarcoma, alveolar rhabdomyosarcoma, and synovial sarcoma, and give a perspective for future developments.

**Graphical abstract:**
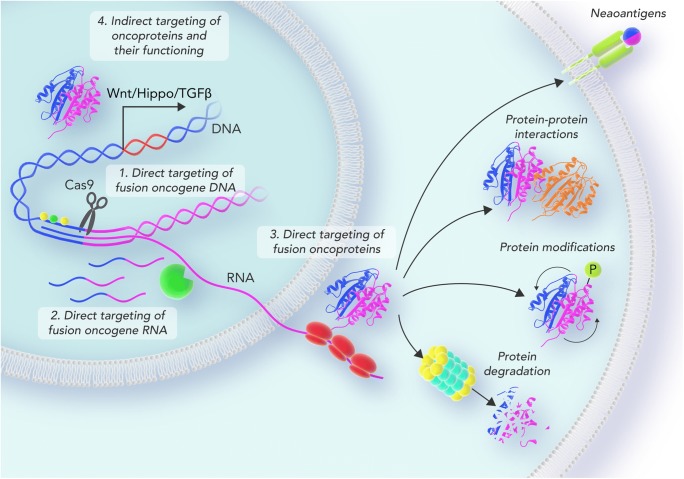
Scheme depicting the different targeting strategies of fusion oncogenes in pediatric fusion-driven sarcomas. Fusion oncogenes can be targeted on their DNA level (1), RNA level (2), protein level (3), and by targeting downstream functions and interaction partners (4).

Scheme depicting the different targeting strategies of fusion oncogenes in pediatric fusion-driven sarcomas. Fusion oncogenes can be targeted on their DNA level (1), RNA level (2), protein level (3), and by targeting downstream functions and interaction partners (4).

## Introduction

Decades after the discovery of *BCR-ABL1* t(9;22)(q34;q11) in chronic myeloid leukemia [1, 2] and *EWSR1-FLI1* t(22;11)(q24;12) in Ewing sarcoma (EwS) [3], more than 20,000 fusion genes have been identified in human malignancies [4]. In cancer entities with high numbers of somatic mutations, the vast majority of gene fusions are supposed passenger mutations, i.e., byproducts of spontaneous genomic rearrangements, which accumulate progressively over time [5, 6]. However, in oligo-mutated childhood cancers, such as EwS, fusion genes are acquired early in tumorigenesis either through balanced chromosomal translocations [3] or through a complex, but well-orchestrated, genomic rearrangement called chromoplexy [7]. Once the respective fusion has occurred, oligo-mutated cancer cells show a strong oncogene addiction toward their disease-defining fusion oncogene (e.g., *EWSR1-FLI1* in EwS [3], *PAX3/7-FOXO1* in alveolar rhabdomyosarcoma (ARMS) [8, 9] and *SS18-SSX1/2/4* in synovial sarcoma (SS) [10–12]) in terms of tumor progression and metastasis.

EwS is the second most common bone cancer in children and was first described by James Ewing in 1921 [13]. It is characterized by a small-round-blue cell phenotype and mostly arises in the metadiaphyseal bones of the lower extremities and in the pelvic region [14]. While EwS is curable in localized disease by radical surgery, radiotherapy, and chemotherapy (5-year survival rate: around 83%), patients with disseminated disease have dismal outcome (5-year survival rate: 37% or less) [15]. Molecularly, EwS is defined by the fusion of the *EWSR1* gene and a transcription factor of the *ETS*-family, such as *EWSR1-FLI1* (85%) or *EWSR1-ERG* (10%) [14]. This fusion event rewires the affinity of the DNA-binding domain of FLI1 and enables it to bind to GGAA-microsatellites (GGAA-mSats) in addition to its physiological binding to the ETS-specific DNA motif ACCGGAAGT. Interestingly, the affinity to those mSats correlates strongly with an increasing number of GGAA-repeats [16]. In turn, binding of EWSR1-FLI1 to such GGAA-mSats converts them into *de novo* enhancers and super-enhancers [16, 17]. Subsequently, EWSR1-ETS fusion oncoproteins deregulate the gene expression of hundreds of genes, such as *MYBL2* [18] and *PPP1R1A* [19], which contribute to the malignant phenotype of EwS. Notably, germline variants of GGAA-mSats that affect the repeat length have recently been reported to contribute to EwS susceptibility and tumor progression in EwS [18, 20].

Alveolar rhabdomyosarcoma (ARMS) is a subtype of rhabdomyosarcoma that is believed to originate from precursor cells in musculoskeletal tissue or mesenchymal stem cells (MSCs) [21] and affects mostly children and adolescents [22]. Histologically, ARMS resembles the architecture of lung tissue by forming fibrovascular septa segregating the small, often discohesive growing tumor cells in an alveoli-like pattern [22]. Approximately two thirds of ARMS harbor a fusion oncogene, whereas one third is fusion oncogene negative [23]. In fusion-positive ARMS, the FOXO1 transactivation domain is fused to the DNA-binding domain of either PAX3 or PAX7 [8, 9]. In analogy to EwS, the generated fusion oncoprotein binds a unique DNA motif (ACCGTGACTAATTNN for PAX3-FOXO1) and hijacks this sequence as a *de novo* enhancer [24], which drives the expression of pro-tumorigenic genes. While *PAX3-* and *PAX7-FOXO1*-positive ARMS have the same prognosis in their localized states, *PAX3-FOXO1*-positive tumors show a more aggressive phenotype once they have metastasized [25].

Among fusion oncogene-driven sarcomas, SS is one of the most prevalent ones accounting for approximately 7–8% of all sarcomas [26]. Historically, SS has been believed to originate from synovial cells due to its histopathological appearance and its mostly joint-associated localization [27]. Although the incidence of this tumor entity peaks at the age of 30 years, 30% of the patients are children or adolescents. On a molecular level, SS is characterized by the *SS18-SSX1, SSX2,* or *-SSX4* fusion gene (hereafter collectively referred to as *SS18-SSX*) [10–12]. These fusion proteins have been found to interfere with normal BRG1-associated factor (BAF) complex (alias SWI/SNF complex) formation by competing with wild-type SS18, thereby causing concomitant loss of BAF47, a tumor suppressive subunit of the BAF complex [28, 29]. Subsequently, the altered BAF complex binds broad polycomb domains, opposes PRC-mediated repression of gene sets, and activates transcription of a SS-specific gene signature [29, 30]. Furthermore, it was shown that SS18-SSX fusion proteins directly interact with the Wnt-associated transcription factor family TCF/LEF and histone deacetylases (HDACs) allowing Wnt-ligand independent Wnt-activation in SS cells [31].

While the respective driver mutations in each of the entities outlined above have long been identified, targeted therapies exploiting (fusion) oncogene addiction in these tumors remain to be integrated into standard of care treatment regimens. Given the young age of disease onset, effective yet gentle alternatives to mutilating surgeries and aggressive radio-chemotherapies are urgently needed. In this article, we will review the current literature on fusion oncogene specific therapeutic targets, evaluate their clinical potential, and propose new strategies targeting oncogene dependence in EwS, ARMS and SS.

## Direct targeting of fusion oncogene DNA

### CRISPR-CAS9-based strategies targeting the breakpoint of fusion oncogenes and regulatory elements bound by fusion oncoproteins

Gene engineering experiences a renaissance since the development of the clustered regularly interspaced short palindromic repeats (CRISPR) associated protein 9 (CRISPR-CAS9) technology. CRISPR-CAS9 has been successfully employed in basic and preclinical research for the last couple of years and faces its first phase I clinical trial at the moment [32, 33]. Besides monogenic disorders, malignant tumors driven by a single-driver mutation represent favorable future indications for the therapeutic use of CRISPR-CAS9 [34]. Among them, tumors harboring a fusion oncogene constitute excellent candidates for oligonucleotide-based treatment strategies due to the sequence specificity of their breakpoint region. Recently, Chen et al. showed the efficacy of a CRISPR-CAS9-based strategy inserting a suicide gene specifically into the breakpoint region of two fusion oncogenes in prostate cancer [35]. Similar strategies have been employed in fusion oncogene addicted sarcomas as well. Mitra et al. have recently shown subtotal tumor clearance of subcutaneous EwS xenografts in mice treated with CD99-targeting nanoparticles carrying *EWSR1-FLI1*-specific sgRNA-RNPs [36]. In a human myoblast model, the CRISPR-CAS9 mediated knockout of *PAX3-FOXO1* abrogated colony formation *in vitro* [37]. Another elegant strategy was pursued by Johnson et al., who deleted the GGAA-mSat regulating the expression of *NR0B1* using CRISPR-CAS9 and thereby impaired the proliferation and oncogenic transformation of EwS cell lines [19]. Moreover, the advent of CRISPRi/a technology has enabled researchers to silence or activate DNA regions, respectively, in a highly specific way. Boulay et al. have employed this method to directly silence various GGAA-mSats and could abrogate tumor growth *in vivo* by targeting a *SOX2*-regulating GGAA-mSat-enhancer using CRISPRi [38]. Taken together, CRISPR-CAS9-based strategies represent novel, highly specific therapeutic approaches to hijack oncogene activity by targeting either the fusion gene itself or its respective DNA-binding motif.

### Pharmacologic targeting the epigenetic regulation of fusion oncogene expression

The concept of epigenetic regulation of gene expression and its implications on health and disease, especially cancer, has been widely accepted in the field of basic and translational research [39, 40]. Epigenomic alterations are common in cancer and have become therapeutically accessible by the advent of epigenome-modifying drugs such as HDAC inhibitors [39]. Although the functional principles of the fusion oncogenes in the above-mentioned sarcoma entities have been studied extensively, their transcriptional regulation has not been fully elucidated yet. However, in EwS, a strong dependence on the presence of BRD4 (a member of the bromodomain and extraterminal domain (BET) family) for efficient transcription of EWSR1-FLI1 has been observed [41]. BRD4 is known to bind acetylated lysine residues and promote transcription by interaction with P-TEFb and RNA-polymerase II [42]. Notably, BET inhibition by JQ1 (+) impaired cell viability, clonogenic, and migratory capacity and induced G1-blockage [41]. In ARMS, Entinostat, an HDAC-inhibitor, which has been found to reduce PAX3-FOXO1 expression and induce subsequent chemotherapy sensitization in preclinical models [43, 44], is currently being evaluated in combination with nivolumab in a phase I/II trial (INFORM2) [45] after it was tolerated well in an earlier phase I study (ADVL1513) [46]. As mentioned above, CRISPR-interference, a variant of the CRISPR-CAS9 system that allows the selective silencing of genes and intergenic regions, has been employed to silence GGAA-mSats and thereby disrupt their enhancer capacity in EwS cell lines [38]. This site-specific technology might represent a promising strategy to target fusion oncogenes themselves, not only their binding regions.

## Direct targeting of fusion oncogene RNA

### Targeting mRNA transcripts of fusion oncoproteins using RNA interference (RNAi)

With the advent of the RNAi technique, knockdown of transcripts including mRNAs of fusion oncogenes has become a standard method to investigate their biological role in cell and animal models. Due to their driver function in oncogenesis, direct targeting of fusion oncogene transcripts has been the Holy Grail of fusion-positive pediatric sarcoma treatment. Indeed, knockdown of fusion oncoproteins in EwS [47, 48], ARMS [49, 50], and SS [51, 52] consistently causes G0/G1 cell cycle arrest and cell death *in vitro*, which supports the potential therapeutic usefulness of this approach. Furthermore, it has been observed that suppression of the fusion oncogene or direct downstream target genes can facilitate differentiation of fusion-positive sarcoma cells in combination with adequate differentiation-stimulating media in EwS [53], SS [29, 54], and ARMS [55]. Hence, differentiation induction by RNAi and differentiation inducing drugs (such as LSD1 and HDAC inhibitors [56, 57]) could be therapeutically implemented in pediatric sarcoma treatment regimens, as exemplified by treatment of acute promyelocytic leukemia with all-trans-retinoic acid [58].

Conversely, the therapeutic strategy of direct targeting of fusion oncoprotein transcripts may also have some drawbacks. Indeed, EwS cells in which EWSR1-FLI1 expression was suppressed by RNAi showed higher metastatic potential than those without EWSR1-FLI1 knockdown *in vitro* and *in vivo*, which undermines the rationale for complete therapeutic suppression of fusion oncoprotein transcripts [59]. This phenomenon, however, has been demonstrated, thus far, only in EwS, whereas in SS and ARMS suppression of the respective fusion oncoprotein or their downstream target genes reduces invasion and migration *in vitro* [60–62]. Moreover, high expression of *SLFN1*, one of the EWSR1-FLI1 upregulated genes, has been shown to be associated with higher sensitivity to chemotherapeutics and superior patient survival [63], suggesting the possibility that suppression of EWSR1-FLI1 may lead to the emergence of chemo-resistant phenotype in EwS.

A major obstacle of implementing RNAi technique in the clinic may reside in the difficulty of sufficiently and systemically introducing constructs into tumor cells. Incipient *in vivo* studies using intratumoral application of siRNA demonstrated certain anti-tumor effect in EwS [64, 65]. However, this approach requires frequent intratumoral injection or prior application of siRNA, which is obviously not feasible in the clinical setting. Development of nanoparticles or liposomes enabled systemic application of siRNA in murine xenograft models with mild anti-tumor effects, specifically promoting cytostaticity rather than cytotoxicity, which may be attributed to unsatisfactory suppression of fusion oncoproteins [66, 67]. A more specific approach with siRNA encapsulated into transferrin-conjugated cyclodextrin-containing polycations was tested in a murine metastatic EwS xenograft model, in which the authors exploited the observation that cell-surface transferrin receptors are highly expressed in the TC71 EwS cell line [68]. This study underlines the importance of tumor-specific delivery strategies for efficient RNAi-based therapeutic approaches. Recent advances in understanding exosome functioning and engineering of recombinant exosomes could overcome current limitations by increasing siRNA delivery efficacy [69].

Taken together, we believe that there is still ample room to investigate potential applications of directly targeting fusion oncoprotein transcripts in sarcomas *via* RNAi techniques, which could be facilitated by developing more specific delivery systems and utilizing combinatorial approaches with other therapeutic modalities.

### Targeting the splicing machinery involved in post-transcriptional modification of fusion oncoproteins

The first association of post-transcriptional modifications of transcripts with fusion oncoproteins was identified through investigations on EWSR1-FLI1 function [70]. EWSR1-FLI1 was found to modulate the splicing machinery by interacting with RNA polymerase II [71], non-fused (wild type) EWSR1 [72], and YB1 [73], creating atypical mRNA isoforms. Selvanathan et al. gave an overview for potential isoforms alternatively spliced by EWSR1-FLI1 [74]. Since isoform variations could be a distinct feature of tumor cells, tumor-specific isoform signatures could be therapeutically implemented.

Increased VEGF165/VEGF189 ratio by alternative splicing has been reported to be caused by EWSR1-FLI1 inhibition of CAPER-alpha (RBM39) [75], which confers a more neoangiogenic phenotype to EwS by recruiting bone marrow-derived progenitor cells in a murine xenograft model [76]. Moreover, selective intratumoral suppression of VEGF165 expression by RNAi significantly reduced tumor growth *in vivo* [77]. Although specific protein isoforms might represent druggable targets using isoform-specific antibodies, the clinical relevance of this observation remains unclear.

Wild-type EWSR1 has been shown to alternatively splice the transmembrane domain coding exon of FAS/CD95 receptor [78]. In EwS, EWSR1-FLI1 interferes with alternative splicing of FAS/CD95 by direct interaction with non-fused (wild-type) EWSR1, resulting in exon 6 exclusion and production of a soluble isoform, which exhibits lower FAS-mediated apoptosis compared to the transmembrane form [78]. This observation may imply that suppression of EWSR1-FLI1 by RNAi could promote a shift toward a FAS-mediated apoptotic phenotype.

Moreover, EWSR1-FLI1 reduces the elongation speed of RNA polymerase II on CCND1 leading to preferential alternative splicing of the D1b isoform, which has a higher oncogenic potential than the D1a isoform [79]. Thus, suppression of EWSR1-FLI1 may lower the D1b/D1a ratio, which could sensitize EwS cells toward CDK4/6 inhibitors.

One of the subunits of the chromatin remodeling BAF complex, ARID1A, has been recently shown to be a splicing target of EWSR1-FLI1 [80]. ARID1A-L generated by alternative splicing in EwS cells plays an important role in tumor growth and promotes the stability of EWSR1-FLI1. Although this observation clearly demonstrated that complex mechanisms of (alterative) splicing are operative in EwS, it remains to be illuminated if and how they can be addressed therapeutically.

Evidence indicating a direct association of fusion oncoproteins with alternative splicing in SS and ARMS is still missing. However, the ribonucleoprotein SYT-interacting protein/co-activator activator (SIP/CoAA), which functions as a RNA splicing modulator, was found to bind to the SS18-SSX2 fusion oncoprotein, implying a potential role of SS18-SSX in alternative splicing in SS [81]. Although comprehensive data demonstrating potential tumor-specific isoform variations created by fusion oncoproteins is still missing, recent advances in transcriptome analysis may uncover tumor-specific isoform signatures, which could be further exploited to identify potential oncogenic “isoform-addiction”. In turn, researchers may be able to take a new approach in revealing potential therapeutic targets in terms of tumor vulnerability caused by isoform-addiction.

## Direct targeting of fusion oncoproteins

### Targeting post-translational modification, protein folding, and degradation of fusion oncoproteins

Post-translational modifications, such as phosphorylation, acetylation, or ubiquitination, are a key aspect of the regulation of protein function. These modifications can allow or inhibit the activity of a protein by changing its structure, inducing intracellular redistribution, or marking the protein for degradation [82]. In the case of the above-mentioned fusion oncoproteins, pharmacological alteration of their post-translational modifications is an interesting strategy to therapeutically influence these oncoproteins, as they are difficult to target directly.

In ARMS, phosphorylation of PAX3-FOXO1 at multiple sites has been shown to be important for stability of the fusion oncoprotein and its transcriptional activity [83]. In accordance with this, the multikinase inhibitor PKC412 has been reported to reduce the phosphorylation of certain sites at the PAX3 domain, to decrease the transcriptional activity of PAX3-FOXO1, and to exert an anti-tumorigenic effect *in vitro* and in xenograft models [83, 84]. As a more specific example, Polo-like kinase 1 (PLK1), a serine/threonine kinase, also phosphorylates and stabilizes the fusion protein [85]. In contrast, the authors could demonstrate that phosphorylation of wild-type FOXO1 at the equivalent serine residue triggers nuclear export of the transcription factor [85]. Inhibition of PLK1 with the small molecule BI 2536 causes increased proteasomal degradation of PAX3-FOXO1 and tumor regression in a xenograft mouse model, an effect that could be reproduced in a different study using the PLK1 inhibitor Volasertib [85, 86]. Furthermore, multiple studies have shown PAX3-FOXO1 to be a substrate of GSK3β, inhibition of which reduces transcriptional activity of the fusion protein and proliferation of tumor cells in a PAX3-FOXO1 dependent manner [87, 88]. Additionally, PAX3-FOXO1 is phosphorylated by cyclin-dependent kinase 4 (CDK4) in the ARMS cell line RH30 and inhibition of CDK4 by facsaplysin reduces the transcriptional activity and increases its cytoplasmatic levels, indicating redistribution [89]. In contrast, evidence has been published that phosphorylation of PAX3-FOXO1 by AKT reduces transcription of PAX3-FOXO1 target genes [90]. Concordantly, indirect activation of AKT *via* the SERCA inhibitor thapsigargin has been shown to reduce binding of PAX3-FOXO1 to regulatory DNA elements and to reduce proliferation *in vitro* and *in vivo* [91].

EWSR1-FLI1, the most common fusion oncoprotein in EwS, has also been found to be subject to various post-translational modifications. Evidence of phosphorylation of EWSR1-FLI1 was first gathered in 2001 by Olsen and Hinrichs, who identified a PKC phosphorylation site and reported this phosphorylation to be essential for transcriptional function of the oncoprotein [92]. Interestingly, PKC-β has been recognized to be necessary for EwS growth in xenograft experiments and a decrease in PKC-β activity might be a factor responsible for the reduced DNA-binding and transcriptional activity of EWSR1-FLI1 and the cytotoxic effect observed after treatment with Englerin A [93, 94]. Additionally, a different site of EWSR1-FLI1 has been shown to be a target of MAPK/ERK and JNK (c-Jun N-terminal kinase) after DNA damage but the functional and potential therapeutic relevance of these phosphorylations remains to be elucidated [95]. Similarly, evidence of acetylation, methylation by PRMT1/PRMT8, and O-GlcNAcylation of EWSR1-FLI1 has been reported with yet mostly unclear functional and therapeutic implications [96–99]. Recently, Gierisch et al. identified EWSR1-FLI1 to be subject to ubiquitination and subsequent proteasomal degradation [100]. Furthermore, they recently demonstrated that the ubiquitin-specific protease 19 (USP19) deubiquitinates the fusion oncoprotein [101]. Depletion of USP19 was shown to decrease EWSR1-FLI1 expression levels, but neither the expression of wild-type of EWSR1 nor FLI1, and to reduce tumor growth *in vitro* and *in vivo* indicating a potential therapeutic value for USP19 inhibition [101]. In addition to that, EWSR1-FLI1 has been reported to be a substrate of the chaperone HSP90 and inhibition of HSP90 by the small molecule PU-H71 reduced tumor cell growth *in vitro* and tumor burden in xenograft experiments [102]. On a similar note, it has been shown that reduced levels of EWSR1-FLI1 can also be achieved by treatment with the HDAC1/3 inhibitor Entinostat, as HDAC1/3 deacetylates HSP90 and thereby increases its activity [103].

Even though the fusion oncoprotein in SS was identified over 25 years ago and there are multiple sites of post-translational modification present in the domains of wild-type SS18 and SSX1/2/4, little has been published about the impact of post-translational modifications on SS18-SSX and their therapeutic value [10, 11]. Nevertheless, Patel et al. have recently shown that SS18-SSX is ubiquitinated and thereby marked for degradation by the MCL-1 ubiquitin ligase E3 (MULE) at the lysine K23 of SS18, which is not a known ubiquitination site in wild-type SS18 [104]. Furthermore, they showed that MULE is a substrate of the E3 ubiquitin-ligase MDM2, which is only active in its deacetylated state that is ensured by the histone deacetylase 2 (HDAC2) [104–106]. As a reduced activity of HDAC2 thus leads to increased ubiquitination and degradation of SS18-SSX, this might be an additional mechanism responsible for the cytotoxic effect of HDAC inhibition in SS cells [107].

### Targeting fusion oncoprotein-specific neoantigens by immunotherapy

The successful clinical implementation of checkpoint inhibitors and CAR-T cells in the routine treatment of various tumor entities has heralded a new era of immunotherapies in cancer [108, 109]. Tumor-specific neoantigens derived from fusion oncogene breakpoint regions have attracted great scientific interest for many years, especially as an alternative to single-nucleotide variant-derived tumor-specific antigens [110]. Recently, gene-fusion-derived neoantigens were found to induce tumor-specific T cells and facilitated a complete response in a head and neck-cancer patient treated with immune checkpoint inhibitors despite an overall low tumor mutational burden [111]. Hence, fusion oncogene-driven pediatric sarcomas might represent promising candidates for immunotherapies targeting breakpoint-derived neoantigens.

Indeed, breakpoint-specific neoantigens have been predicted and in part validated for EWSR1-FLI1 [112], PAX3-FOXO1 [112], and SS18-SSX1 [113]. For EWSR1-FLI1, it was shown that a modified 9-mer peptide of the fusion site (YLNPSVDS) was able to induce a robust immune response *in vitro* and *in vivo* facilitating prolonged survival of xenograft-bearing mice after treatment with YLNPSVDS-specific cytotoxic T lymphocytes (CTLs) [114]. This is of special interest, since native peptides of the breakpoint region have failed to provoke significant CD8^+^ CTL responses before and show weak MHC-binding affinities [114, 115]. Moreover, fusion-restricted immune responses seem to highly depend on the HLA alleles. For instance, two highly similar peptides of the PAX3-FOXO1 breakpoint region showed strikingly different results depending on the HLA alleles used in the respective study: The RS10 peptide (SPQNSIRHNL) was able to induce highly effective CTLs in HLA-B7^+^ donor peripheral blood mononuclear cells and allowed killing of HLA-B7^+^ ARMS cells [116]. In contrast, the modified 9-mer peptide GLSPQNSIK, which shares 6 amino acids with RS10 and was optimized for HLA binding, failed to induce a potent CTL response in HLA-A3^+^ CD8^+^ T cells [117]. Targeting neoantigens derived from the breakpoint region of fusion oncogenes represents a sophisticated, yet challenging approach. The development of CAR-T cells specific for the peptide-MHC-complex might represent a promising strategy to tackle the low antigenicity of fusion peptides in the future.

## Indirect targeting of fusion oncoproteins and their function

### Targeting protein-protein interactions of fusion oncoproteins

Fusion oncogenes and their function have been extensively characterized over the past decades. By now, it has been well accepted that fusion oncogenes orchestrate a tumorigenic re-programming comprised of a plethora of interaction partners rather than acting in isolated systems. This holds especially true for SS18-SSX oncoproteins that exert their deleterious effects by interacting with the BAF complex, a multimember epigenetic regulatory machinery [29]. Such interaction partners might represent druggable targets that could indirectly compromise fusion oncoprotein functioning.

Apart from their direct DNA-binding capability, EWSR1-FLI1 and PAX3-FOXO1 also act through direct protein-protein interactions. For instance, it has been shown that EWSR1-FLI1, but not wild-type EWSR1, directly binds RNA helicase A [118] and reduces helicase activity [119]. RNA helicase A is involved in many cellular processes such as re-organizing RNA secondary structures, spliceosome assembly, and initiation of translation. In turn, blocking this interaction using the compound YK-4-279 translated in increased apoptosis and reduced growth of EwS orthotopic xenografts and might represent an elegant strategy to target this neomorphic feature of the fusion oncogene [120]. Moreover, Li et al. found a direct link between p53 inhibition and EWSR1-FLI1 [121]. Mutations in p53 have been found in as little as 5–7% of EwS [14]; however, even in wild-type sarcomas, p53 function seems to be compromised in analogy to other fusion-positive malignancies such as fusion-driven leukemia [121–123]. In EwS, the N-terminal domain of EWSR1-FLI1 was identified to form a complex with HDAC1 and p53 leading to subsequent deacetylation of p53 and inhibition of its downstream signaling [124]. However, it remains unclear whether this interaction is indeed a neomorphic feature of the fusion oncogene and not intrinsic to wild-type EWSR1, especially since the interaction seems to involve the N-terminal region of EWSR1-FLI1 [121, 124].

As outlined before, a recent study revealed a direct interaction of EWSR1-FLI1 with the BAF complex *via* its subunit ARID1A [80]. The authors could show that ARID1A was differentially spliced and accumulated in its long isoform ARID1A-L in the presence of EWSR1-FLI1. Notably, modulation of wild-type EWSR1 levels did not affect the isoform switch, pointing toward a neomorphic feature of the fusion gene [80]. ARID1A-L was shown to stabilize EWSR1-FLI1 and bolstered its oncogenic capacity. Interestingly, the above-mentioned compound YK-4-279 also blocked this interaction and prevented binding of EWSR1-FLI1 to the BAF complex *via* ARID1A. Hence, TK216, a slightly improved version of YK-4-279 that has been approved for a phase I clinical trial in relapsed or refractory EwS, represents a very promising candidate for future targeted therapies of this devastating disease [125].

Lastly, Embree et al. could demonstrate the interaction of EWSR1-FLI1 with wild-type EWSR1 in a zebrafish model and in human cell lines [72]. The researchers observed mitotic defects in EWSR1-FLI1 expressing zebrafish embryos and HeLa cells. Since the observed phenotype closely resembled one that was observed in EWSR1-deficient embryos, a direct interaction of EWSR1-FLI1 with EWSR1 was suspected. Indeed, it was found that the fusion oncoprotein bound wild-type EWSR1 and inhibited its function resulting in spindle malformation and mis-localization of Aurora kinase B [72]. Anyhow, the authors did not expand on how these findings might translate into therapeutic consequences and the effects of targeting EWSR1 in EwS still remain to be investigated.

Apart from EWSR1-FLI1, PAX3-FOXO1 functioning has also been found to rely on direct interactions with other proteins, especially kinases, as reviewed above [85–91]. Although all of the phosphorylation sites also exist in wild-type PAX3 or FOXO1, respectively, and thereby do not represent neomorphic features of the fusion oncogene *per se*, kinase inhibitors showed different effects on the fusion oncogene than on wild-type PAX3 and FOXO1 [85]. Moreover, some of them directly interfered with its binding to the neomorphic binding site ACCGTGACTAATTNN that is enriched in PAX3-FOXO1 associated super-enhancers [91]. Hence, kinase inhibitors do not particularly exploit neomorphic features rather than inhibiting neomorphic functioning of PAX3-FOXO1.

In SS, the interactions of its fusion oncoprotein with the BAF complex have been widely studied and are reviewed below. Besides subunits of the BAF complex, only a few other proteins have been demonstrated to bind SS18-SSX fusion proteins directly. For instance, SS18-SSX1 was found to stabilize HDM2, a negative regulator of p53 that consecutively promoted p53 ubiquitination and degradation resulting in resistance to apoptosis-inducing drugs [126].

To conclude, targeting proteins that directly interact with fusion oncoproteins might constitute a useful addition to well-established therapies or novel treatment strategies directed against the fusion oncoprotein itself. Yet, the lower specificity of such approaches and the resulting side effects might constitute an obstacle for future clinical trials.

### Targeting protein-DNA interactions of fusion oncoproteins including epigenetic rewiring and subcellular localization

While EWSR1-FLI1 and PAX3-FOXO1 are aberrant transcription factors directly binding to specific DNA sequences, SS18-SSX instead interacts with chromatin remodeling complexes [14, 22, 28, 30, 127]. Therefore, the oncogenicity of these oncoproteins is rooted in their influence on the state of chromatin and on the expression of certain genes. Preventing these oncoproteins to access the DNA might consequently be a promising strategy for more specific therapies of these sarcomas.

As an example, the small molecule lurbinectedin and trabectedin have been found to induce a nucleolar redistribution of EWSR1-FLI1 in EwS cells leading to an increase in heterochromatin-associated histone methylations at EWSR1-FLI1 target genes [128, 129]. Both compounds have preclinically shown efficacy against EwS cells, especially in combination with other compounds such as irinotecan or olaparib [128, 130]. Lurbinectedin also has shown a good safety profile and anti-cancer activity as a single agent in a phase II clinical trial [131].

As fusion-driven sarcomas are characterized by the paucity of other genetic mutations and due to the functions of their disease-defining oncogenes, epigenetic dysregulation plays an important role in the maintenance of their phenotype. Hence, epigenetic modulators, which are increasingly becoming of interest in other malignancies as well, are major potential targets in EwS, SS and ARMS.

One group of epigenetic regulators are HDACs, which remove acetyl groups from lysine residues of histone chains and thereby regulate gene expression [132]. Indeed, in preclinical models of EwS, HDAC inhibitors have been shown to induce apoptosis, promote differentiation, and reduce tumor growth in xenograft experiments [57, 133]. Antitumorigenic activity of HDAC inhibitors has also been reported in preclinical models of SS and ARMS [134–138]. Unfortunately, the few clinical trials that have been conducted to investigate the efficacy of HDAC inhibition have shown only modest response at best in these entities, when used as single agents [139–143]. HDAC inhibitors might therefore be of greater use in combination with other anti-cancer drugs. Apart from standard chemotherapeutics, possible combination partners might also include additional epigenetic drugs. Using the single agent SP2509, reversible inhibition of lysine-specific demethylase 1 (LSD1), a subunit of the Mi-2/nucleosome remodeling and deacetylase (NuRD) complex, induces apoptosis and reduces xenograft tumor growth in EwS [144]. Interestingly, this LSD1 inhibitor has a synergistic effect with the HDAC inhibitors suberoylanilide hydroxamic acid (SAHA) or romidepsin *in vivo* [145, 146]. Based on these preclinical studies, more investigations focusing on this and other combinations with HDAC inhibitors are justified.

Another interesting epigenetic modulator is the enhancer of zeste homolog 2 (EZH2), a Polycomb-group protein involved in DNA methylation [147]. Knockdown or pharmacological inhibition of EZH2 in ARMS cells leads to apoptosis and reduced tumor growth of ARMS xenografts *in vivo* [148]. In EwS, the EZH2 promoter is a direct target of EWSR1-FLI1. Here, RNAi mediated downregulation of EZH2 inhibits clonogenicity *in vitro* and suppresses tumor development and metastasis in xenograft experiments [149]. However, a tumor xenograft study using 4 different cell lines of ARMS and EwS showed only limited anti-tumor activity of the EZH2 inhibitor tazemetostat for both entities [150]. Therefore, further preclinical and clinical investigation into the use of EZH2 inhibitors for treatment of ARMS and EwS is of need. In SS cells, inhibition of EZH2 with the small molecule EPZ005687 has been shown to impair proliferation and migration [151]. However, in a phase II clinical trial of tazemetostat as a single agent in patients with SS, stable disease could be achieved in only 33% of treated patients indicating the possible necessity of combination partners [152].

Yet, another group of proteins involved in chromatin regulation is the bromodomain and extra terminal domain (BET) family. Its members, such as BRD4, are able to recognize and bind acetylated histones with their bromodomains and play a role in epigenetic memory and transcription regulation [153]. With the advent of specific BET bromodomain inhibitors (JQ1 and iBET) bromodomain containing proteins have entered the spotlight as attractive potential targets for a variety of different cancer entities [154, 155]. In EwS cells, BRD4 has been found to form a transcriptional complex with EWSR1-FLI1 and -ERG. RNAi mediated knockdown or JQ1 mediated inhibition of BRD4 resulted in significantly impaired oncogenic phenotype and reduced xenograft tumor growth [156]. ARMS cells have also been reported to be sensitive to JQ1 in preclinical models [157]. In addition to that, Gryder et al. could explain this sensitivity mechanistically by showing that PAX3-FOXO1 function relies on BRD4 recruitment at defined super-enhancers [24]. Furthermore, it was recently demonstrated that BRD9 is a component of the SS18-SSX containing BAF complexes in SS [158]. Inhibition or targeted degradation of BRD9 induced downregulation of oncogenic programs and reduced growth of SS xenografts [158]. In the light of these preclinical results, clinical trials to investigate the efficacy of BET inhibitors for the treatment of fusion-driven sarcomas as single agents or in combinations seem to be warranted.

### Targeting specific downstream pathways of fusion oncoproteins

Malignant transformation and maintenance of tumorigenesis in EwS, SS, and ARMS depend on the expression of their specific fusion proteins to the extent that their deletion results in cell death. However, there are no available drugs that can directly target these fusion proteins, so far. Since the expression of these fusion proteins also completely rewires the expression and regulation of pathways and downstream effectors, there is great potential for alternative targeted therapeutic interventions. This section will cover a range of targeted therapies described to date against commonly altered oncogenic pathways in these fusion-driven sarcomas.

#### Insulin-like growth factor receptor (IGF-1R) pathway

Tumorigenesis in translocation-driven tumors is critically mediated *via* the IGF-1R signaling pathway [159]. Preclinical EwS models show a constant activation of the IGF-1R-mediated signaling pathway [160], which promotes drug escape [161] and seems to be essential for EwS cell viability [162]. In SS, the translocation induces the expression of IGF2, which is also required for tumor growth *in vivo* [163], while its immune targeting inhibits tumor growth and metastasis formation in ARMS [164]. Additionally, IGF-1R inhibition induces apoptosis of SS cells *in vitro* [165]. At the same time, IGF-1R is a direct target of the PAX3-FOXO1 fusion in ARMS [166].

Several antibodies and small molecules against IGF-1R pathway have been tested in clinical trials including phase II and III. However, the results have only been moderate so far due to acquired resistance through different mechanisms [167–169]. Yet, given the outstanding preclinical results obtained when blocking this pathway, significant efforts are being made to develop combined therapies that could overcome these hurdles. For instance, a recent report suggests combining heparanase inhibitors with IGF-1R antagonists for treatment of metastatic SS [170], and a combined therapy including BET inhibitors has been set forth for EwS [171]. In the case of ARMS, it has been proposed that the simultaneous inhibition of IGF-1R and additional tyrosine kinases could help to overcome resistance to treatment. For instance, concomitant inhibition of IGF-1R and YES/SRC family tyrosine kinase [172], a combination of an anti-IGF-1R antibody and the inhibition of the anaplastic lymphoma kinase (ALK) [173], or treatment with Ceritinib (ALK and IGF-1R inhibitor) with the multikinase inhibitor sorafenib have been suggested based on preclinical results [174].

#### Mitogen-activated protein kinase pathway

Tyrosine kinases are highly expressed in sarcomas including EGFR and PDGFRA in SS, FGFR, and EGFR in ARMS, and upstream mediators of ERK1/2 in EwS [175, 176]. Indeed, ERK1/2 is activated in the majority of EwS cell lines and is thought to hold a pivotal role in tumor development [177]. In fact, the A673 EwS cell has a constitutively activating mutation in *BRAF* [178]. Accordingly, the inhibition of ERK1/2 precursor MEK1/2 using U0126 impedes migration and invasion of EwS cell lines [177]. Besides, pharmacological treatment with FGFR inhibitor PD-173074 yielded a decrease in tumorigenic features in preclinical studies [179]. Therefore, mitogen-activated protein kinase (MAPK) pathway blocking agents such as RAF inhibitors have entered clinical trials [180].

In SS, the situation is partially different: although ERK1/2 MAPK signaling pathway components have been proposed as targets of SS18-SSX, few reports describing their role and druggability in SS are available so far [181, 182]. However, there is a targetable upregulation of EGFR, PDGFRA, and PDGFRB in SS that can be exploited therapeutically by using specific small molecule inhibitors [183, 184]. Additionally, and similarly to EwS, FGFR2 is also being studied as a potential therapeutic target in SS [185]. Finally, it has been recently shown that nintedanib, a triple kinase inhibitor targeting PDGFR, VEGFR, and FGFR pathways, presents promising effects in a preclinical study for SS that are beyond those shown by imatinib [186]. In ARMS, the upregulation of EGFR presents a rationale for the use of anti-EGFR monoclonal antibodies such as cetuximab that has proven to be effective in preclinical studies [187]. Notably, due to the high expression of FGFR4, the inhibitor PD-173074 has shown similar preclinical promising effects in ARMS as in EwS [176]. Moreover, sorafenib, a PDGFRA and RAF inhibitor, reduced tumor growth in a mouse model for ARMS [188].

#### Sonic hedgehog (SHH) pathway

In ARMS, the expression of sonic hedgehog pathway effectors like GLI1/2 is altered [189]. Inhibitory targeting of GLI1/2 using GANT-61 leads to a blockage of tumor growth *in vivo*, and its effect was shown to be synergistic with commonly used chemotherapeutic drugs for ARMS such as temsirolimus or vincristine [190]. Similarly, a decrease in cell viability and induced cell death was obtained *in vitro* by treatment with arsenic trioxide (ATO), a different GLI1 inhibitor [191], and *in vivo* by treatment with the sonic hedgehog pathway inhibitor foskolin [192]. Additionally, apoptosis-inducing agents like betulinic acid have been demonstrated to impair tumor growth in preclinical studies *via* inhibition of the hedgehog pathway in ARMS [193]. Finally, in order to avoid escape mechanisms, targeted therapies including GANT61 and the dual PI3K/mTOR inhibitor PI103 have shown a synergistic effect on ARMS cells apoptosis [194].

GLI1 was also shown to be upregulated in EwS and driven by the translocation [195], and therefore, therapeutic approaches using ATO have been attempted with promising preclinical results [196]. Thus, ATO was included in the Pediatric Preclinical Testing Program (PPTP) [197] where it unfortunately yielded negative results.

In the case of SS, since *GLI* and *SMO* were found to be overexpressed in patient’s tumors [198] and since Notch has been shown to activate GLI1, a Notch inhibitor in combination with vismodegib was tested in phase II trial that accepted SS patients to be enrolled (NCT01154452). Unfortunately, the inhibitor was discontinued and the trial had to be prematurely ended.

#### Wnt/β-catenin pathway

The Wnt pathway is activated in most SS [199, 200] and this activation is mediated by SS18-SSX [201]. Following this rationale, Wnt inhibitors such as pyrvinium have been preclinically tested with promising results [202]. Beyond blocking the pathways using SS18-SSX-directed small molecules, targeting some of its interacting proteins has been attempted. For instance, desatinib-a YES1 tyrosine kinase inhibitor-has been demonstrated to block the Wnt pathway leading to impairment of cell proliferation in SS [203]. Additionally, SS patients have been enrolled in a results-pending trial to test an antibody against the Wnt receptor FZD10 (NCT01469975).

In contrast, the relevance and potential for clinical intervention in the Wnt pathway in ARMS remains highly controversial [204]. So far, a single study by Annavarapu et al. found an inhibition of proliferation and self-renewal capacity after pharmacological re-activation of the Wnt pathway *in vitro* [205].

Interestingly, in EwS, even though Wnt activation is consistent with worse clinical outcomes and a more aggressive phenotype *in vitro*, cell populations present a highly heterogeneous activation of the Wnt signaling pathway, which could be at least partially mediated by the inhibition of EWSR1-ETS protein functions [206]. So far, WNT974-a modulator of the Wnt pathway at PORCN level-showed a significant delay in formation of metastasis *in vivo* [207].

All in all, combined targeting of oncogenic fusion proteins along with specific pharmacological intervention of one or multiple deregulated pathways may constitute a promising therapeutic approach to overcome drug resistance for these fusion-driven malignancies.

## Conclusions and perspectives

**Fig. 1:**
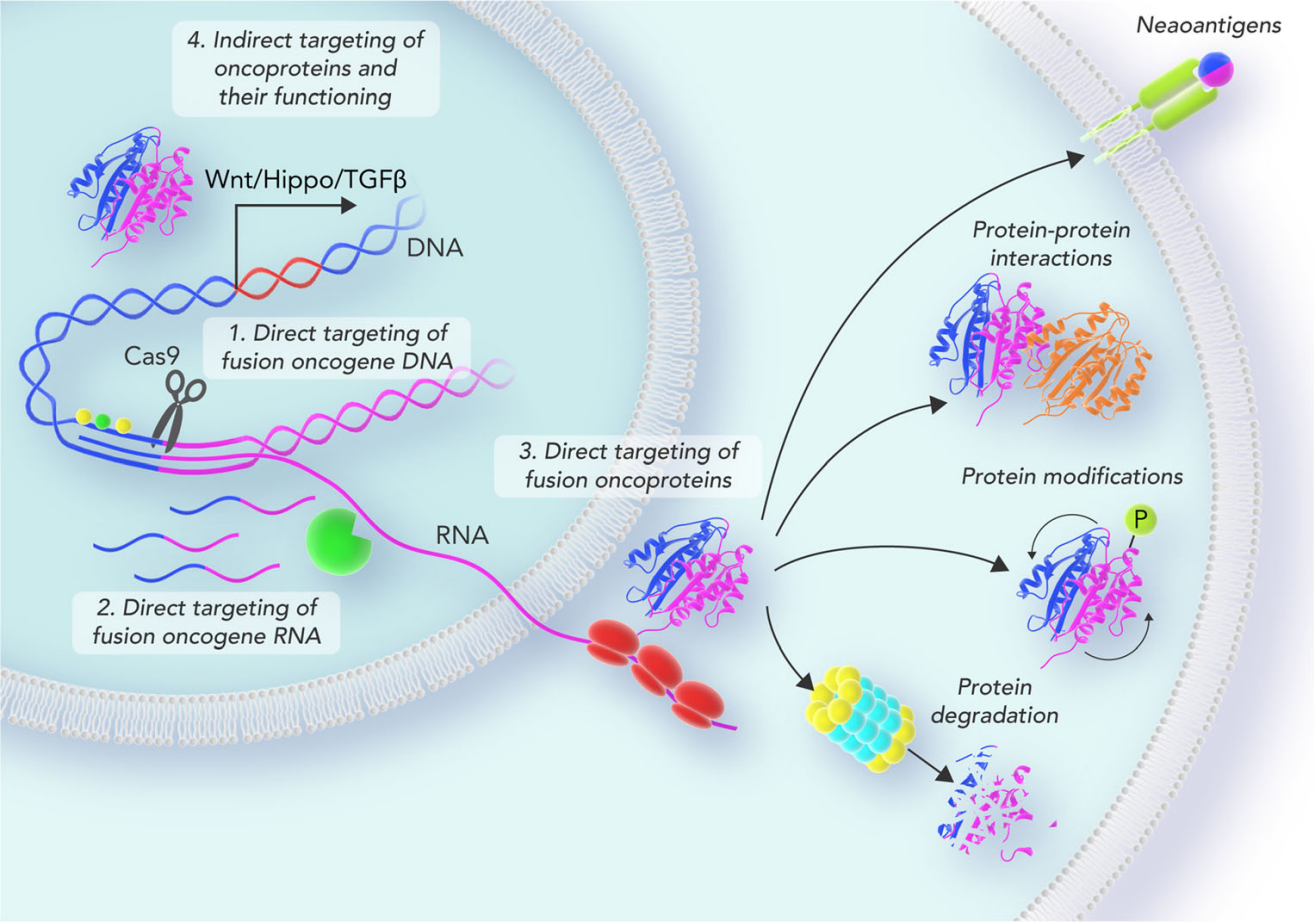
Scheme depicting the different targeting strategies of fusion oncogenes in pediatric fusion-driven sarcomas. Fusion oncogenes can be targeted on their DNA level (1), RNA level (2), protein level (3), and by targeting downstream functions and interaction partners (4).

Reviewing the current literature on neomorphic features of fusion oncoproteins in EwS, ARMS, and SS, we have presented four modalities of targeting fusion oncogenes in these devastating childhood sarcomas (Figure 1). First, oncogene DNA or their respective binding sites can be directly targeted by gene editing strategies, such as CRISPR-CAS9 or CRISPRi. Second, oncogene mRNA transcripts represent a favorable target for RNAi-based therapeutic approaches. Moreover, fusion oncoproteins that interfere with the splicing machinery allow isoform-specific treatment strategies. Third, oncoproteins and their subcellular localization can be directly targeted either by inhibiting or inducing their post-translational modification, respectively. Additionally, breakpoint regions constitute excellent targets for immunotherapies once they are sufficiently presented by the tumor cells. Fourth, unique interactions of fusion proteins with their respective interaction partners have already been successfully exploited in clinical trials (e.g., using YK-4-279). Furthermore, due to the lack of somatic mutations and the importance of epigenetic alterations for the malignant phenotype of fusion-driven sarcomas, targeting epigenetic regulators has been found to be promising in these entities. In a more system-based manner, tackling downstream pathways and regulatory networks of fusion oncogenes might add to the arsenal of indirect treatment options in EwS, ARMS and SS.

Taken together, neomorphic features of EWSR1-FLI1, PAX3/7-FOXO1, and SS18-SSX fusion proteins have been therapeutically addressed in the past and represent a potent, highly specific approach worthy of further investigations in the future. New delivery strategies, advances in understanding and exploiting tumor immunity, new drugs targeting fusion oncoproteins, epigenetic regulators, and interaction partners might help to overcome the hurdles that have proved these oncogenes to be notoriously difficult to target.
